# C-type Lectin CD209L/L-SIGN and CD209/DC-SIGN: Cell Adhesion Molecules Turned to Pathogen Recognition Receptors

**DOI:** 10.3390/biology10010001

**Published:** 2020-12-22

**Authors:** Nader Rahimi

**Affiliations:** Department of Pathology, School of Medicine, Boston University Medical Campus, Boston, MA 02118, USA; nrahimi@bu.edu

**Keywords:** CD209, L-SIGN, CD209L, DC-SIGN, C-type lectin, cell adhesion molecule, C-type lectin domain family 4 member M, CLEC4M, LSECtin, CLEC4G, SARS-CoV-2, COVID-19

## Abstract

**Simple Summary:**

COVID-19 pandemic continues to pose a serious threat to global public health with overwhelming worldwide socio-economic disruption. SARS-CoV-2, the viral agent of COVID-19, uses its surface glycoprotein Spike (S) for host cell attachment and entry. The emerging picture of pathogenesis of SARS-CoV-2 demonstrates that S protein, in addition, to ACE2, interacts with the carbohydrate recognition domain (CRD) of C-type lectin receptors, CD209L and CD209. Recognition of CD209L and CD209 which are widely expressed in SARS-CoV-2 target organs can facilitate entry and transmission leading to dysregulation of the host immune response and other major organs including, cardiovascular system. Establishing a comprehensive map of the SARS-CoV-2 interaction with CD209 family proteins, and their roles in transmission and pathogenesis can provide new insights into host-pathogen interaction with implications in therapies and vaccine development.

**Abstract:**

C-type lectin CD209/DC-SIGN and CD209L/L-SIGN proteins are distinct cell adhesion and pathogen recognition receptors that mediate cellular interactions and recognize a wide range of pathogens, including viruses such as SARS, SARS-CoV-2, bacteria, fungi and parasites. Pathogens exploit CD209 family proteins to promote infection and evade the immune recognition system. CD209L and CD209 are widely expressed in SARS-CoV-2 target organs and can contribute to infection and pathogenesis. CD209 family receptors are highly susceptible to alternative splicing and genomic polymorphism, which may influence virus tropism and transmission in vivo. The carbohydrate recognition domain (CRD) and the neck/repeat region represent the key features of CD209 family proteins that are also central to facilitating cellular ligand interactions and pathogen recognition. While the neck/repeat region is involved in oligomeric dimerization, the CRD recognizes the mannose-containing structures present on specific glycoproteins such as those found on the SARS-CoV-2 spike protein. Considering the role of CD209L and related proteins in diverse pathogen recognition, this review article discusses the recent advances in the cellular and biochemical characterization of CD209 and CD209L and their roles in viral uptake, which has important implications in understanding the host–pathogen interaction, the viral pathobiology and driving vaccine development of SARS-CoV-2.

## 1. Introduction

Lectins are a diverse family of carbohydrate recognizing proteins that possess carbohydrate-recognition domain (CRD) or sulfated glycosaminoglycan (SGAG)-binding motif [1,2]. Derived from the Latin word “legere”, meaning “to select”. Lectins were originally identified for their selective carbohydrate binding properties. However, now it is known that they can also mediate protein-protein, protein-lipid or protein-nucleic acid interactions [3]. By virtue of their CRD, lectins have the ability to recognize specific carbohydrate structures on proteins, which in turn, mediate cell-cell and cell-pathogen interactions [4,5]. There are currently fourteen structural families and three related subfamilies of lectins in human genome that span 76 different genes [6,7]. The C-type (calcium-dependent) lectins with 66 gene members is one of the largest subgroups of the lectin superfamily [6,8] that are further separated into multiple subgroups [7,9,10]. One of these subgroups is the CD209/DC-SIGN (Dendritic cell-specific ICAM-3-grabbing non-integrin 1 also called CLEC4L, C-type lectin domain family 4 member L) subgroup, which includes CD209/DC-SIGN (and three other member genes, namely CD209L/L-SIGN/CLEC4M (Liver/lymph node-specific ICAM-3-grabbing non-integrin, C-type lectin domain family 4 member M), CD23 and LSECtin/CLEC4G [11,12]. Mouse genome encodes five homologues of human CD209 with a variable sequence homology to human CD209 [13], but it is not clear whether their function is similar to human CD209L and CD209. Other major lectin subfamily proteins are the P-type lectins (mannose 6-phosphate (M6P) and the I-type lectins. Siglecs (Sialic acid-binding immunoglobulin-type lectins) are the best characterized I-type lectins [14,15]. Given the role of CD209L and related proteins in diverse mechanisms of pathogen recognition and emerging evidence for the role of CD209 family proteins in SARS-CoV-2 entry and infection [16], this review article particularly has focused on the recent advances in the cellular and biochemical characterization of CD209 and CD209L and their roles in virial uptake, which could provide valuable insights pertinent to current pathobiological studies and therapeutic development of vaccines for SARS-Cov-2.

## 2. CD209/DC-SIGN and CD209L/L-SIGN Family Proteins: Cell Adhesion Molecules Turned to Pathogen Recognition Receptors:

The conserved physiological function of CD209 family proteins is to mediate cell-cell adhesion by functioning as high affinity receptors for intercellular adhesion molecules 2 and 3 (ICAM2 and ICAM3/ CD50) [17,18]. CD23 acts as a low-affinity receptor for immunoglobulin E (IgE) and CR2/CD21 [19] and LSECtin interacts with CD44 on activated T cells [20]. A survey of current literature indicates that these receptors are also among the most common pathogen recognition receptors present in the human genome [21,22]. CD209L and CD209 serve as receptors for Ebolavirus [23], Hepatitis C virus [24], human coronavirus 229E [25], human cytomegalovirus/HHV-5 [26], influenza virus [27], West-Nile virus [26], Dengue virus [28] and Japanese encephalitis virus [29]. Recently, we and others have shown that CD209 and CD209L is capable of recognizing SARS-CoV and SARS-CoV-2 [16,26,30,31]. In addition to its ability to recognize a plethora of viruses, CD209 is also known to recognize parasites such as leishmania amastigotes [32] and Yersinia pestis coccobacillus bacterium [33]. The complete list of viruses that are recognized by CD209, CD209L and LSECtin are shown (Table 1). To date, it is not known whether CD23 is involved in any pathogen recognition.

It is increasingly evident that viruses exploit host lectin receptors like the CD209L family proteins and others for two major reasons; to promote infection of target cells and evade the immune recognition system. In many cases lectin receptors such as CD209 and CD209L are employed as functional portals for viral recognition and infection. However, in some other cases, they may also enable infection of target cells via trans-infection (i.e., cell captures the pathogen without entry and then passes it to another cell, which is also a replication-independent mechanism [50]. For example, CD209 expressed in DCs can bind to HIV envelope glycoprotein, gp120, without triggering cell-virus fusion [51]. The interaction of CD209 with gp120 appears to be complex as it can lead to both positive and negative outcomes for virus, perhaps depending to cell type in which CD209 is expressed. In some cases, CD209-captured virions are internalized and targeted to the lysosome for degradation [52,53]. However, in cases which HIV-1 receptor and co-receptors (CD4 and CCR5/CXCR4) are present on the host cells, CD209 can facilitate infection by transferring the virus to immune cells [54,55]. Additionally, it was found that CD209-dependent capture of HIV-1 virions could transiently protect virions from degradation, which ultimately leads to viral infectivity [17,56,57,58], suggesting that CD209 positive DCs capture and internalize HIV-1 virions and homes them to lymph nodes [59]. However, the Trojan horse model of HIV transmission by CD209 was challenged by various studies [60]. Studies on B lymphocytes and platelets indicate that CD209 expressed in these cells successfully mediate the entry of and infection by HIV-1 [61,62,63]. Similarly, CD209 and CD209L interact with Ebola virus glycoprotein and mediate infection of endothelial cells via both cis- and trans-infection [23,36]. Likewise, the recent findings also support for CD209L-mediated cis- and trans-infection of SARS-CoV-2 [16,64]. Aside from the role of CD209 and CD209L in cis- and trans-infection and transmission, the recognition of these receptors by pathogens also can impact the host defense mechanism against these pathogens. For instance, CD209-dependent viral entry and infection can initiate signaling events in host cells that compromise immune responses and promote infection of DCs [65,66]. Interestingly, although CD209L and CD209 are devoid of any enzymatic activity, however upon interaction with pathogens, they can stimulate activation of multiple protein kinases, GTPases and phosphatases [67].

## 3. CD209/DC-SIGN and CD209L/L-SIGN Family Proteins and Coronaviruses:

The evolutionarily conserved mechanism by which human coronaviruses including, CoV-229E, NL63, OC43, HKU1, MERS-CoV, SARS-CoV, SARS-CoV-2 recognize the host cells rely on the viral glycoprotein spike (S) that interacts with specific receptors on the host target cells. Intriguingly, the S protein appears to be highly adept and can interact with different types of host receptors. For examples, the S protein of CoV-229E and transmissible gastroenteritis virus (TGEV) employ CD13 (aminopeptidase N) as a receptor for entry and infection of target cells [68,69], whereas S protein of CoV-NL63 and HKU1 interact with glycan-based receptors carrying 9-O-acetylated sialic acid (9-O-Ac-Sia) [70,71]. The S protein of MERS-CoV uses Dipeptidyl peptidase 4 (DPP4/CD26) and carcinoembryonic antigen-related cell adhesion molecule 5 (CEACAM5) as attachment or entry receptors for infection [72,73]. CEACAM5 appears to facilitate MERS-CoV infection by enhancing the attachment of the virus to the host cell surface [73]. The S protein of Filoviridae Marburg virus, SARS-CoV [26,31] and SARS-CoV-2 [16,74] can employ the carbohydrate-recognition domain (CRD) containing CD209L and CD209 lectins as attachment or entry receptor.

CD209L is broadly expressed in human lung, kidney epithelium and endothelium [16]. Furthermore, human endothelial cells are permissive to SARS-CoV-2 infection and interference with CD209L activity via shRNA or soluble CD209L inhibited SARS-CoV-2 entry and replication [16]. Remarkably, the S protein of human coronaviruses including, NL63 [75], SARS-CoV [76] and SARS-CoV-2 [16] can also employ angiotensin-converting enzyme 2(ACE2) as an entry receptor for infection, suggesting that both ACE2 and the lectin family proteins, CD209L and CD209, contribute to the spread of these pathogens in vivo. Previous studies on SARS-CoV demonstrated a direct role for CD209L and CD209 in infection by acting as entry receptors for SARS independent of ACE2 [77,78,79,80]. Curiously, CD209L can physically interact with ACE2 [16], suggesting both ACE2-dependent and independent mechanisms for CD209L-mediated viral entry. However, the underlying mechanism of CD209L and CD209 mediated SARS-CoV-2 infection is not fully understood and requires further investigation.

## 4. Topology of CD209L/L-SIGN and CD209/D-SIGN

**Figure 1:**
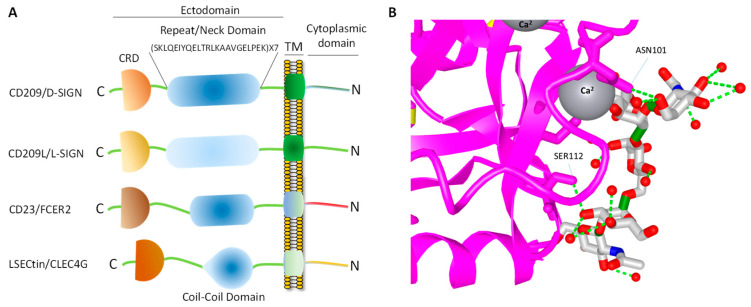
CD209 family proteins. (**A**) Graphic presentation of CD209 family proteins and the key domain information. The schematic of domains do not directly correlate to the number of amino acids in each domain. (**B**) Crystal structure of a typical CRD complexed with carbohydrate and the position of the Ca^2+^ ion, which makes a tertiary complex between lectin and carbohydrate structure.

CD209 family proteins are type II transmembrane glycoprotein receptors (*i.e*., C-terminus is exposed outside the lipid bilayer and N-terminus resides in the cytosol). The ectodomain of CD209 and CD209L is composed of the neck region followed by the CRD. These domains represent the most distinct and functional features of these two receptors (Figure 1A). The neck/repeat region is composed of 23 amino acids which is repeated seven times in CD209L and CD209 and three times in CD23/FCER (Figure 1A). However, the neck/repeat region on LSECtin/CLEC4G is replaced with a coil-coil motif, which is also involved in protein-protein interaction (Figure 1A). Central to recognition of cellular and pathogen glycoproteins, is the presence of the CRD on the C-terminus of CD209 family proteins, which is paramount to recognition of mannose containing structures present on specific glycoproteins.

**Figure 2:**
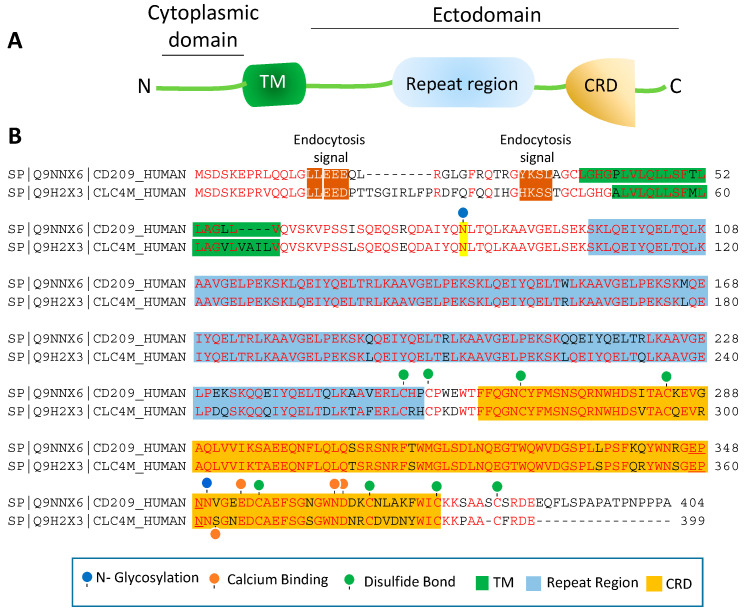
Amino acid sequence homology of CD209 and CD209L: (**A**) The schematic of CD209L is shown. (**B**) Alignment of the amino acids of human CD209 and CLEC4M (gene encoding for CD209L called C-type lectin domain family 4 member M, CLEC4M). The key common features of CD209L and CD209L, including potential PTMs and ion bindings are highlighted.

CRD is a 110–130 amino acid long with a double-looped, two-stranded anti-parallel β-sheet connected by two α-helices and a three-stranded anti-parallel β-sheet [81]. Typically, CRD has two conserved disulfide bonds and up to four Ca^2+^ binding sites, depending on the specific family of lectin. Amino acid residues with the carbonyl side chains are involved in coordinating Ca^2+^ in the CRD, and these residues also directly bind to carbohydrates leading to a ternary complex formation between a carbohydrate in a glycan, the Ca^2+^ ion, and amino acids within the CRD. A typical CRD-carbohydrate interaction is shown (Figure 1B). Amino acid sequence alignment of CD209L with CD209 illustrates that these proteins are highly conserved, suggesting that they likely evolved through gene duplications. There are at least two putative internalization motifs at the cytosolic N-terminus tail of CD209 and CD209L, indicating that both CD209 and CD209L upon interaction with pathogens are capable of undergoing internalization and delivering the pathogen inside the target cells. The internalization motifs are di-leucine (LL) and tyrosine(Y)-based (Figure 2B), but, the key tyrosine residue in the tyrosine-based internalization motif on the CD209L is replaced with histidine (H) (Figure 2B), indicating that CD209L undergoes internalization solely via di-leucine motif [82].

The ectodomain of CD209 and CD209L is composed of the neck region followed by the CRD. These domains also represent the most distinct and functional features of these two receptors. The neck region which is a repeat of 23 amino acids (Figure 2B), is involved in protein dimerization/oligomerization [83,84], and may also contribute to increased pathogen recognition and concentration of pathogens at the cell surface. The neck region forms an α-helical coiled-coil fold that is thought to stabilize the oligomerization of CD20 family proteins [85,86]. The presence of CRD on the ectodomain is paramount to recognition of mannose, fucose- or galactose-containing structures on the pathogens and the cellular ligands by CD209 and CD209L. It is thought that within the CRD a highly conserved EPN motif (Glu-Pro-Asn) is responsible for recognition of mannose, fucose- or galactose-containing structures [87]. Yet, despite a high degree of homology of the amino acid residues in the CRD of CD209L and CD209, there is evidence for differential recognition of oligosaccharide structures by these receptors. For example, CD209L appears to prefer mannose oligosaccharides but not fucose-containing carbohydrates such as LewisX (LeX) glycans [88]. Interestingly, a recent analysis revealed that *N*-glycosylation of SARS-CoV-2 spike protein is largely oligomannose-type glycans [89], which may account for the strong binding of SARS-CoV-2 spike protein with CD209L and CD209 [16]. Furthermore, while the ectodomain of CD209L contains two *N*-glycosylation sequons, at sites N92 and N361 (Figure 2B), only N92 is occupied.

Curiously, removal of *N*-glycosylation on the CD209L increases the binding of CD209L with the SARS-CoV-2 spike protein [16], suggesting that *N*-glycosylation of CD209L may generate a hindrance for the CRD-mediated glycoprotein interaction [16] and may have impact in virus tropism and transmissibility in vivo. A similar hindering mechanism for ligand-receptor interaction by *N*-glycosylation was reported for an unrelated receptor tyrosine kinase, vascular endothelial receptor-2 (VEGFR-2) interaction with its ligand [90]. Another important, yet poorly understood aspect of CD209 family proteins is their cytoplasmic N-terminus domain, which is vital for their signal transduction relays. To date, there is no evidence for potential posttranslational modifications (PTMs) or a direct protein interaction between the cytoplasmic N-terminus domains of CD209L and CD209 with the signaling proteins [67]. Unlike many of their counterpart receptors, the cytoplasmic N-terminus domains of CD209L and CD209 contain no conserved immunoreceptor tyrosine-based inhibitory (ITIM, V/IXYXXL/I/V) motif, which interacts with the Src-homology 2 (SH2) domain containing proteins [91]. While, the cytoplasmic N-terminus domains of CD209L contains no tyrosine residue, the cytoplasmic N-terminus domain of CD209 contains one tyrosine residue with a weak sequence homology to ITIM motif (Figure 2B). But, there is no experimental evidence whether the key tyrosine (Y31) residue is phosphorylated and recruits any SH2 domain signaling proteins to CD209. Furthermore, there are multiple serine/threonine residues (four on the CD209 and 8 on the CD209L) on the cytoplasmic N-terminus domains of CD209L and CD209 (Figure 2B), which potentially could be phosphorylated. Similarly, there are multiple lysine (K) residues on the cytoplasmic N-terminus domain of CD209L and CD209 with potential to undergo ubiquitination. In particular, K5, which is conserved both in CD209L and CD209, has a high probability to be ubiquitinated. Ubiquitin modification regulates both proteolytic and non-proteolytic functions of proteins [92].

## 5. Decoy CD209L and CD209 Proteins:

**Figure 3:**
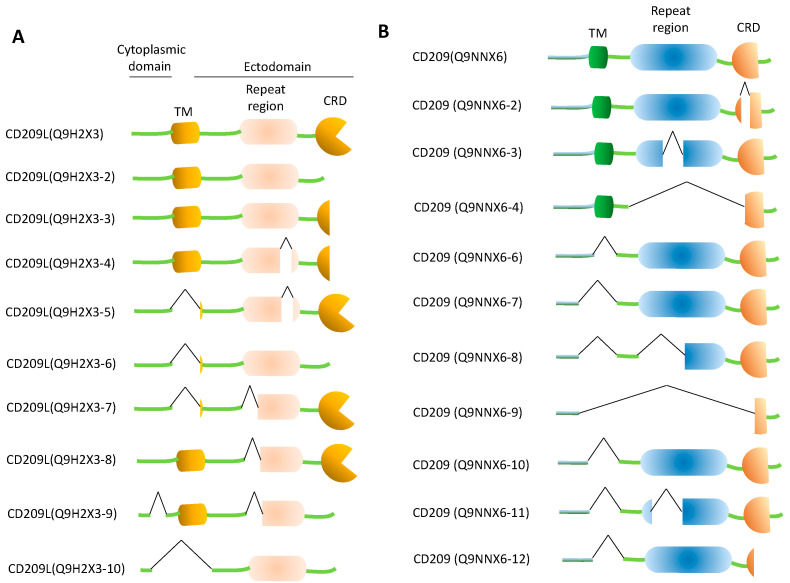
The schematics of alternatively spliced variants of CD209L and CD209. Amino acid sequences of alternatively spliced variants of CD209L (**A**) and CD209 (**B**) were aligned via Clustal Omega software program. The schematic of each alternatively variant proteins were presented.

A corollary to the function of CD209L and CD209 in pathogen and cellular ligand recognition is that these receptors are highly susceptible to alternative splicing and genomic polymorphism, which may significantly influence their core functions. Analysis of CD209L via uniprot (https://www.uniprot.org/), a freely accessible resource of protein sequence, revealed that CD209L mRNA can generate at least 9 alternatively spliced variants (Figure 3A). In many cases, the CRD is either completely or partially deleted (Figure 3A), which generates a carbohydrate binding decoy of CD209L. In some other cases, the transmembrane domain is deleted, which results in the soluble form of CD209L. Yet in other cases, multiple deletions occur simultaneously leading to generation of soluble CD209L proteins without CRD (Figure 3A). Some of the alternatively spliced variants of CD209L also carry a deletion on the neck region (Figure 3A). Similar to CD209L, CD209 also due to alternative splice mechanism can also yield 11differant variants (Figure 3B). However, the tissue expression profiles and the potential function of these alternatively spliced variants in normal cellular ligand recognition, and more importantly in pathogen interaction remains largely unknown. Moreover, various recent studies have shown a distinct genomic polymorphism, specifically, in the tandem-neck-repeat region of *CD209L* [93,94] and on the promoter *CD209* [95], which is linked to the pathogenesis of tuberculosis [96] and resistance to HIV-1 [97]. These polymorphic changes most likely influence the physiological function of CD209L family proteins as well as the role that CD209L family proteins play in binding to other pathogens, like SARS and SARS-CoV-2.

## 6. Expression Profile of CD209L Family Proteins in Human Tissues and Cells:

**Figure 4:**
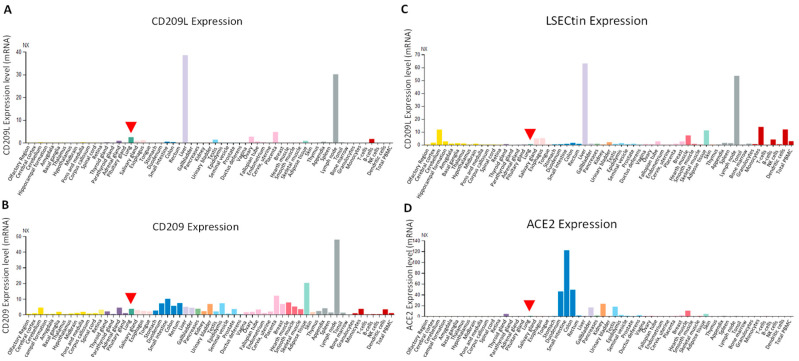
Expression profile of CD209L family proteins and ACE2. (A-D) Data were extracted from the Human Expression Atlas (data was accessed in 11/03/2020) [101]. Arrowhead points to expression of individual genes in lung.

Survey of the published data indicates that CD209/DC-SIGN is predominantly expressed on the monocyte-derived dendritic cells (mo-DCs), and on mo-DCs of immature and mature in lymphoid tissue, lymph nodes and spleen [18,98], whereas CD209L is predominantly expressed in human type II alveolar cells of lung, liver, kidney and lymph nodes [16,18,31,99]. Our analysis of expression of CD209L, CD209 and LSECtin mRNAs through the Human Expression Atlas, a publically available dataset [100], revealed that CD209L expression is relatively restricted to a few human organs. The highest levels of CD209L was observed in liver and lymph node followed by placenta, lung and ovary (Figure 4A). CD209, on the other hand, is broadly expressed in human tissues and organs at the various levels. The highest levels of CD209 expression was observed in the lymph node followed by the adipose tissue, small intestine and rectum (Figure 4B). Similar to CD209, LSECtin/CLEC4G was also broadly expressed in human tissues and organs. The highest levels of LSECtin was observed in liver and lymph node, followed with DCs, monocytes, adipose tissue, heart muscle and cerebellum (Figure 4C). Considering the current SARS-CoV-2 pandemic, it is worthwhile to compare the expression profiles CD209 family proteins with ACE2. Human ACE2 is considered an important entry receptor for SARS-CoV-2 [76,101,102] and was previously reported to be widely expressed in the lung, vascular system and other organs [103]. However, a recent study demonstrated that ACE2 is expressed at very low levels and only in a small subset of lung epithelial cells [104] and low-to-undetectable levels in endothelial cells [105]. In agreement with the recent observations, our analysis of ACE2 mRNA through the Human Expression Atlas revealed that ACE2 is highly expressed only in intestinal tissues (small intestine, colon and duodenum) and at the low levels in the hearth muscles, kidney, gallbladder and testis, but undetectable in lung (Figure 4D). The observed limited/low ACE2 expression pattern in human tissues suggests that SARS-CoV-2 could use alternative receptors for cell entry in a cell-type dependent manner as CD209L and CD209 appear to be more broadly expressed in human organs and tissues than the ACE2. Given the wider tissue expression patterns of CD209 and CD209L in SARS-CoV-2 target tissues, they could play important roles in the pathogenesis of SARS-CoV-2.

## 7. SARS-CoV-2, CD290 Family Proteins and Endothelial Cells:

**Figure 5:**
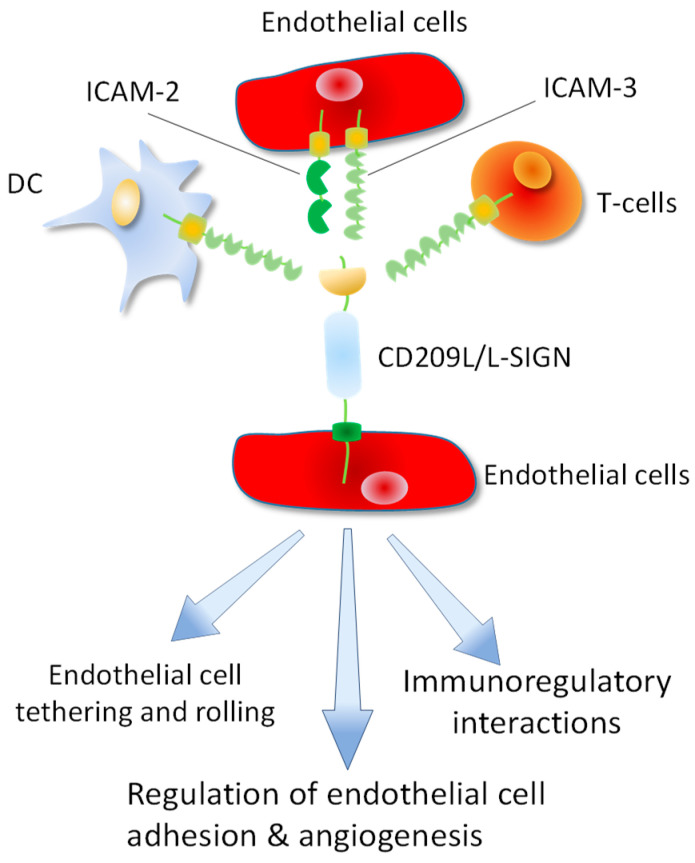
CD209L/L-SIGN mediates multiple cell-cell interactions between endothelial cells, DCs and T-cells. CD209L cellular ligands (ICAM2 and ICAM3) are expressed in DCs, T-cells and endothelial cells leading multiple immunoregulatory and other interactions between T-cells, DCs and endothelial cells. CD209L/ICAM2/ICAM3 interactions in endothelial cells can regulate endothelial adhesion and angiogenic properties of endothelial cells.

CD209L is expressed on endothelial cells of various organs including, the lymph nodes sinuses [106], liver sinus endothelial cells [106], capillary endothelial cells of the placenta, the endothelial cells of the gastrointestinal tract [107], kidney endothelial cells [16] and pulmonary endothelial cells [16,31,108]. In addition, other CD209L family lectin, LSECtin/CLEC4G is also expressed in liver sinusoidal endothelial cells and in the lymph node [12]. CD209L expressed in endothelial cells can interact with ICAM3 which is expressed in DCs, T-cells [109,110] and endothelial cells [18,111] generating multiple immunoregulatory interactions between T-cells, DCs and endothelial cells (Figure 5). Moreover, endothelial cells express both ICAM3 [18,111] and ICAM2 [112], capable of generating homotypic interactions between endothelial cells (Figure 5) that regulate endothelial function and angiogenesis. ICAM2 and ICAM3 belong to immunoglobulin (Ig) superfamily cell adhesion molecules. ICAM2 and ICAM3 are highly glycosylated at the ectodomain and contain two and five Ig domains, respectively. Consistent with this idea, loss of ICAM2 in mouse results in impaired endothelial cell migration and angiogenesis [112]. However, expression of ICAM2, ICAM3 and CD209L in endothelial cells, the role of this CD209L/ICAM2/ICAM3 axis in endothelial biology is largely unknown. A recent study demonstrated that knocked down of CD209L by shRNA in immortalized endothelial cells increased cell migration, but inhibited capillary tube formation/in vitro angiogenesis [16], underscoring the importance of CD209L pathway in the regulation of normal endothelial function.

Recent studies demonstrate that human vascular system is a direct target of SARS-CoV2 [16,113,114]. Although primarily infects the lungs, the SARS-CoV-2 virus also targets multiple other organs including, the cardiovascular system, gastrointestinal tract, and the kidneys [115,116,117,118]. Severe endothelial cell injury, vascular thrombosis with micro-angiopathy, occlusion of alveolar capillaries, and angiogenesis are commonly observed in COVID-19 patients [114]. COVID-19 patients suffer from distinct endothelial cell injury (*i.e*., endothelitis), and altered angiogenesis with widespread microvascular thrombosis [119,120]. These observations coupled with the fact that vascular endothelial dysfunction also plays crucial roles in the pathogenesis of COVID-19 [22,121], underscores the pivotal role of endothelial cells in the pathobiology of SARS-CoV-2 infection. Collectively, the data points to potentially important role for CD209 family proteins in the pathogenesis of SARS-CoV-2. Therefore, CD209L not only can act as a SARS-COV-2 entry receptor, but also performs critical functions in the angiogenic responses of endothelial cells, suggesting that SARS-COV-2, by exploiting CD209L, could undermine CD209L function in endothelial cells, leading to endothelial cell injury and altered angiogenesis.

## 8. Conclusions and Perspective:

Infectious diseases have been foremost among the threats posed to human health and survival through history. The novel coronavirus disease-2019 (COVID-19) pandemic continues to pose a serious threat to global public health with overwhelming worldwide socio-economic disruption. The emerging picture of pathogenesis of SARS-CoV-2 is that in addition to dysregulation of the host immune response in lung, also targets other major organs including, cardiovascular system and others, which may account for COVID-19 induced mortality. The vascular system, particularly, the pulmonary endothelium may play a pivotal role in the pathogenesis of COVID-19 via engagement of CD209L and other related receptors. To date, many aspects of SARS-CoV-2 transmission, infection, and treatment remain unclear. Not only can CD209L function as an entry receptor it can also contribute to the pathogenesis of COVID-19. Establishing a comprehensive map of the SARS-CoV-2 interaction with CD209 family proteins, and their roles in endothelial functions and injury can provide new insights into pathogenesis of COVID-19 and offers a bona fide treatment modality.

## Figures and Tables

**Table 1:** List of known pathogens recognized by CD209, CD209L and LSECtin lectin family proteins. The data is extracted from the publications available through PubMed.

Gene Name	Pathogen Name	References
CD209	HIV-1 and HIV-2	[34,35]
	Ebolavirus	[36,37]
	Cytomegalovirus	[24,38]
	Hepatitis C virus	[24]
	Dengue virus	[28]
	Measles virus	[39]
	Herpes simplex virus	[40]
	Influenza virus A	[27]
	SARS-CoV-2	[16]
	SARS-CoV	[30]
	MERS	[41]
	Japanese encephalitis virus	[29]
	Lassa virus	[42]
	Respiratory syncytial virus	[43]
	Rift valley fever virus	[44]
	Uukuniemi virus	[44]
	West-Nile virus	[45]
CD209L	Ebolavirus	[23]
	Hepatitis C virus	[24]
	HIV-1	[37,46]
	Human coronavirus 229E	[25]
	Human cytomegalovirus/HHV-5	[47]
	Influenza virus	[27]
	SARS-CoV	[26]
	SARS-CoV-2	[16]
	West-Nile virus	[26]
	Japanese encephalitis virus	[29]
	Marburg virus	[26]
LSECtin	Japanese encephalitis virus	[12]
	Ebolavirus	[48]
	SARS-CoV	[48]
	Lassa virus	[49]
